# Evidence that Proteasome-Dependent Degradation of the Retinoblastoma Protein in Cells Lacking A-Type Lamins Occurs Independently of Gankyrin and MDM2

**DOI:** 10.1371/journal.pone.0000963

**Published:** 2007-09-26

**Authors:** Ryan T. Nitta, Catherine L. Smith, Brian K. Kennedy

**Affiliations:** Department of Biochemistry, University of Washington, Seattle, Washington, United States of America; University of Hong Kong, China

## Abstract

**Background:**

A-type lamins, predominantly lamins A and C, are nuclear intermediate filaments believed to act as scaffolds for assembly of transcription factors. Lamin A/C is necessary for the retinoblastoma protein (pRB) stabilization through unknown mechanism(s). Two oncoproteins, gankyrin and MDM2, are known to promote pRB degradation in other contexts. Consequently, we tested the hypothesis that gankyrin and/or MDM2 are required for enhanced pRB degradation in *Lmna^−/−^* fibroblasts. *Principal Findings.* To determine if gankyrin promotes pRB destabilization in the absence of lamin A/C, we first analyzed its protein levels in *Lmna^−/−^* fibroblasts. Both gankyrin mRNA levels and protein levels are increased in these cells, leading us to further investigate its role in pRB degradation. Consistent with prior reports, overexpression of gankyrin in *Lmna* ^+/+^ cells destabilizes pRB. This decrease is functionally significant, since gankyrin overexpressing cells are resistant to p16^ink4a^-mediated cell cycle arrest. These findings suggest that lamin A-mediated degradation of pRB would be gankyrin-dependent. However, effective RNAi-enforced reduction of gankyrin expression in *Lmna^−/−^* cells was insufficient to restore pRB stability. To test the importance of MDM2, we disrupted the MDM2-pRB interaction by transfecting *Lmna^−/−^* cells with p14^arf^. p14^arf^ expression was also insufficient to stabilize pRB or confer cell cycle arrest, suggesting that MDM2 also does not mediate pRB degradation in *Lmna^−/−^* cells.

**Conclusions/Significance:**

Our findings suggest that pRB degradation in *Lmna^−/−^* cells occurs by gankyrin and MDM2-independent mechanisms, leading us to propose the existence of a third proteasome-dependent pathway for pRB degradation. Two findings from this study also increase the likelihood that lamin A/C functions as a tumor suppressor. First, protein levels of the oncoprotein gankyrin are elevated in *Lmna^−/−^* fibroblasts. Second, *Lmna^−/−^* cells are refractory to p14^arf^-mediated cell cycle arrest, as was previously shown with p16^ink4a^. Potential roles of lamin A/C in the suppression of tumorigenesis are discussed.

## Introduction

A-type nuclear lamins are intermediate filament proteins that contribute to the organization and maintenance of the nuclear structure [1], [2]. The A-type lamins (predominantly Lamins A and C, two alternatively spliced products of the *LMNA* gene), along with B-type lamins comprise a protein meshwork underlying the inner nuclear membrane known as the nuclear lamina [3]. A-type lamins also localize to internal regions of the nucleus [4], [5]. Biochemical studies have shown that lamin A/C can interact with different gene regulators including SREBP1 [6], MOK2 [7], and the retinoblastoma protein, pRB [8], [9]. pRB regulates cell cycle progression by inhibiting the activity of the transcriptional activator E2F and through establishment of repressive chromatin structures at genes necessary for S phase entry and progression [10], [11]. When active, hypophosphorylated pRB can arrest cells during the G1 phase of the cell cycle [12], [13]. By regulating expression and/or activity of pRB, A-type lamins can regulate both gene expression and cellular proliferation.

Several lines of evidence indicate that A-type lamins and pRB are physically and functionally linked [14], [15]. For instance, during the G1 phase of the cell cycle hypophosphorylated pRB is anchored to the nuclear matrix by directly binding to lamin A/C and a lamin associated protein, LAP2alpha [8], [16]–[19]. In addition, pRB localizes to internal nuclear foci enriched in lamin A/C [5]. This nuclear tethering is important for pRB function since cells lacking A-type lamins possess decreased pRB levels and resistance to p16^ink4a^-induced G1 arrest [20]–[22], a phenotype associated with cells deficient in pRB [23] . Consistently, reducing expression of LAP2alpha by siRNAs also reduces pRB levels, further supporting a “scaffolding” model in which pRB activity depends on its attachment to lamin A/C or LAP2alpha [24]. In the absence of lamin A/C, pRB is degraded in a proteasome-dependent manner suggesting that A-type lamins protect pRB from degradation. However, the mechanism by which A-type lamins stabilize pRB is currently unknown [21], [22].

Recently an oncoprotein, gankyrin, was shown to interact and stimulate pRB degradation [25], [26]. Gankyrin was identified as the p28 component of the 26S proteasome [27]–[29], and as an overexpressed gene from cDNA libraries from hepatocellular carcinomas (HCC) [25], [30]. Gankyrin was found to alter the phosphorylation status of pRB, leading to increased phosphorylation at specific residues [25]. This altered phosphorylation is reportedly linked to the ability of gankyrin to enter a ternary complex with cyclin D and CDK4, altering CDK4 specificity for serine residues on pRB. Gankyrin also competes with p16^ink4a^ for CDK4 association [31]. The exact mechanism in which gankyrin degrades pRB in a proteasome-dependent manner is unknown. One model posits that gankyrin directly binds to pRB, thereby targeting pRB to the 26S proteasome [32]. Another model suggests that gankyrin may facilitate an interaction between pRB and an E3 ubiquitin ligase, MDM2 [33]. MDM2 is a well-documented oncogene that targets p53 for degradation [34]. MDM2 also interacts with and stimulates proteasome-dependent pRB degradation [35]–[37].

Since gankyrin and MDM2 can target pRB for degradation, either or both proteins may be required for pRB degradation in the absence of A-type lamins. For example, lamin A/C may control gankyrin or MDM2 activity, or lamin A/C may regulate pRB in a manner that keeps it inaccessible from these proteins. To examine these possibilities, we have examined gankyrin and MDM2 function in *Lmna^−/−^* cells. While gankyrin levels are elevated in *Lmna^−/−^* fibroblasts, we were unable to find evidence supporting a role for gankyrin or MDM2 in pRB degradation in this context. These findings suggest that in the absence on A-type lamins, pRB degradation occurs by a third, undefined pathway. Our findings do, however provide further support to the assertion that A-type lamins act as tumor suppressors [38], [39].

## Results

### Gankyrin levels are increased in cells lacking A-type lamins

We previously determined that lamin A/C protects pRB from proteasome-dependent degradation, however, the exact mechanism is still unknown [38], [40]. Two proteins, gankyrin and MDM2, were reported to target pRB for degradation [25], [35]–[37], leading us to ascertain whether either of these proteins are required for pRB degradation in *Lmna^−/−^* cells. For gankyrin, two possibilities were considered. First, cells lacking lamin A/C may have elevated levels or activity of gankyrin, leading to pRB degradation. A second possibility is that pRB is more accessible to gankyrin, perhaps as a result of mislocalization [40], in *Lmna^−/−^* cells.

**Figure 1:**
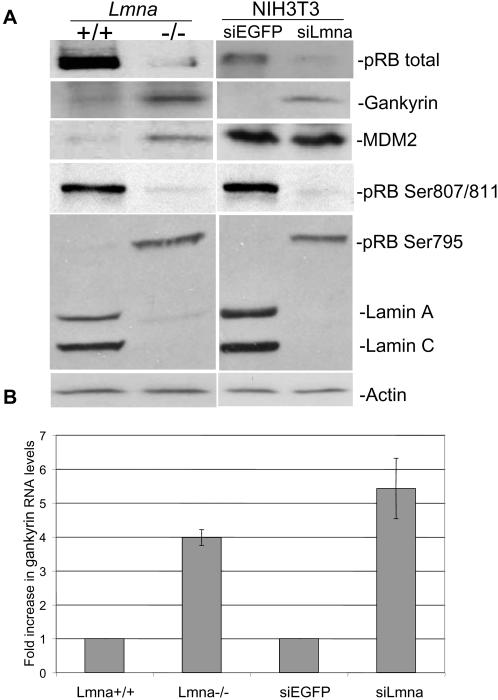
Gankyrin protein and mRNA levels are increased in cells lacking A-type lamins. (*A*) Western analysis comparing relative gankyrin protein levels in cell lines lacking A-type lamins, *Lmna* ^–/–^ fibroblasts and NIH3T3 cells infected with siRNAs targeted to *LMNA* (siLmna) to the control cells, *Lmna* ^+/+^ fibroblasts or NIH3T3 cells infected with siRNAs targeted to enhanced GFP (siEGFP). Actin levels indicate equivalent protein loading here and throughout the manuscript. (*B*) QPCR analysis comparing the relative mRNA's of gankyrin in cells lacking A-type lamins to the control cells. Data represent averages of triplicate experiments performed and mRNAs were normalized against *Gapdh*.

To test if lamin A/C and gankyrin are functionally linked, we initially analyzed gankyrin levels in two different cell lines lacking A-type lamins and their respective controls: (1) immortalized *Lmna^−/−^* fibroblasts [40] and (2) NIH3T3 cells with reduced lamin A/C expression using retrovirally infected siRNAs targeted against *Lmna* (siLmna) or enhanced GFP (siEGFP) [38]. Consistent with our previous findings, total pRB levels are decreased four-fold in cells lacking lamin A/C (*Lmna* ^−/−^ and siLmna cells) compared to the control cells (*Lmna* ^+/+^ and siEGFP cells) [21], [22] (**Figure 1A**). Interestingly, gankyrin protein levels were increased six-fold in cells lacking lamin A/C. Previous studies have shown that cells overexpressing gankyrin have increased pRB phosphorylation at residue Ser 795, but not residues Ser 807 or Ser 811 [25]. Consistently, we found that cells lacking lamin A/C have increased phosphorylated pRB levels at Ser 795, but not Ser 807/811, indicative of increased gankyrin activity (**Figure 1A**). The increase in Ser 795 phosphorylation is especially notable since overall pRB levels are significantly reduced in these cells.

Are gankyrin mRNA levels increased in cells lacking lamin A/C? Quantitative PCR (QPCR) was used to determine whether increased levels of gankyrin correlated with changes in transcript levels. When compared to the control cells (*Lmna* ^+/+^ and siEGFP cells), gankyrin transcript levels were increased by four-fold in *Lmna* ^−/−^ cells and increased five-fold in siLmna cells (**Figure 1B**).

### Reintroduction of lamin A restores gankyrin protein and transcript levels

**Figure 2:**
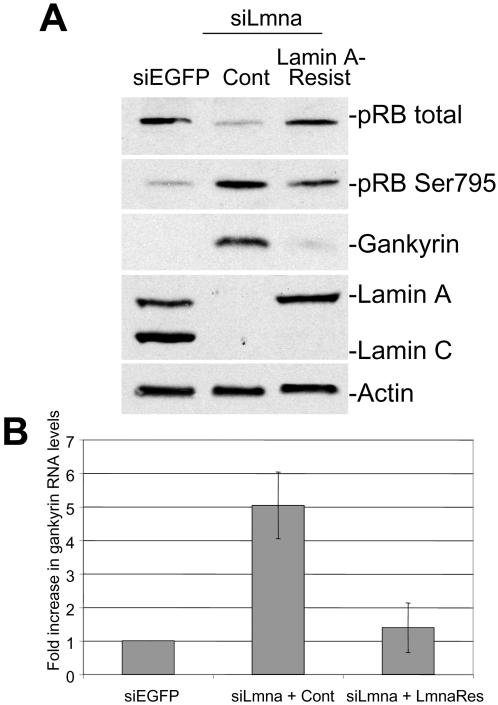
Reintroduction of human lamin A restores pRB and gankyrin expression. NIH3T3 siLmna cells were stably transduced with human lamin A (lamin A-res) that was designed to contain several silent codon changes at the target site of the siRNA, thereby rendering it resistant to the siRNA. (A) Western analysis comparing gankyrin and pRB protein levels in siLmna expressing lamin A-res (siLmna-lamin A resist) to siLmna infected with the control vector (siLmna-Cont). (B) QPCR analysis comparing the relative mRNA's of gankyrin in siLmna-lamin A resist cells to siLmna-Cont cells. Data represent averages of triplicate experiments performed and mRNAs were normalized against *Gapdh*.

To attribute increased gankyrin levels specifically to the loss of lamin A/C, we stably transduced a human lamin A (lamin A-res) that escapes siRNA detection and is therefore resistant to the siRNAs expressed in siLmna cells (see Materials and Methods). Reintroduction of lamin A-res in siLmna cells was sufficient to increase total pRB levels four-fold (**Figure 2A**). This finding was consistent with our previous findings, in which human lamin A was stably transduced in the *Lmna* ^−/−^ cells, resulting in increased pRB levels [22]. We also determined that gankyrin levels were decreased six-fold and pRB Ser795 levels were decreased two-fold indicating that stable lamin A expression was sufficient to restore normal gankyrin levels and activity in siLmna cells. We attribute the observation that pRB Ser795 levels were only reduced two-fold due to the presence of residual gankyrin protein in the lamin A-res cells. Consistently, QPCR analysis showed that reintroduction of lamin A-res in siLmna cells decreased gankyrin transcript levels five-fold compared to the control siLmna cells (**Figure 2B**).

### Overexpression of gankyrin decreases pRB levels and makes cells p16^ink4a^ resistant

**Figure 3:**
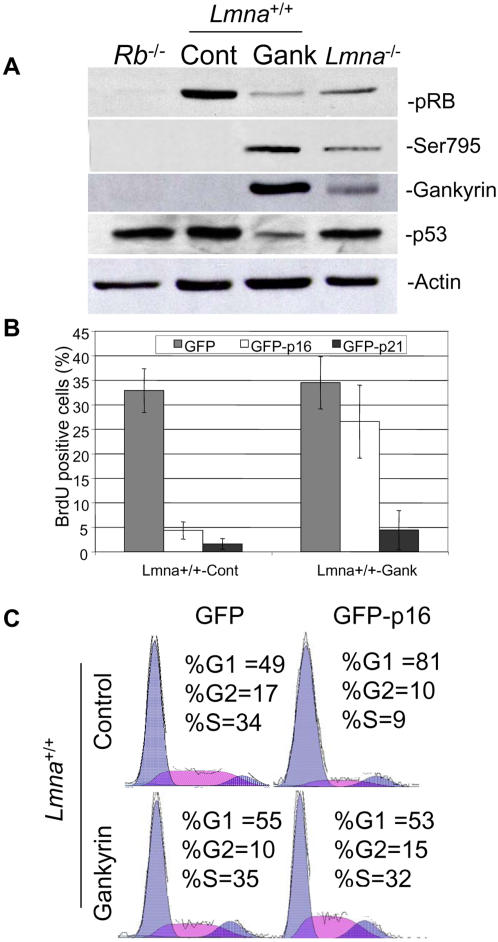
Overexpression of gankyrin reduces pRB levels and makes *Lmna* ^+/+^ cells refractory to p16^ink4a^-induced arrest. (A) Western analysis of *Lmna* ^+/+^ infected with a control plasmid, *Lmna* ^+/+^-Cont, or gankyrin, *Lmna* ^+/+^-Gank, and uninfected *Lmna* ^−/−^ fibroblasts. *Rb* ^−/−^ fibroblasts were included as a control for pRB levels. (B) Cells were transfected with GFP-p16^ink4a^ or GFP-p21^cip1^ and GFP-positive cells were scored; S-phase cells were identified by BrdU incorporation. The percentage of cells positive for GFP and BrdU incorporation were quantified. Results are shown with standard deviations from triplicate samples; 150 cells were counted per sample. (C) Flow cytometry was conducted on *Lmna* ^+/+^-Cont and *Lmna* ^+/+^-Gank cells transfected with GFP or GFP-p16^ink4a^.

It has been reported that overexpression of gankyrin leads to pRB degradation in human osteosarcoma cells [25], a finding we have replicated (**Figure S1**). We generated stable cell lines of *Lmna* ^+/+ ^cells overexpressing gankryin (*Lmna* ^+/+^-Gank) or control vector (*Lmna* ^+/+^-Cont) to determine whether overexpression of gankyrin is also sufficient to destabilize pRB in immortalized mouse fibroblasts. We found that *Lmna* ^+/+^-Gank fibroblasts have six-fold decreased pRB levels compared to *Lmna* ^+/+^-Cont cells (**Figure 3A**). This decrease of pRB was similar to that in *Lmna* ^−/−^ cells indicating that overexpression of gankyrin yielded a similar cellular phenotype as loss of A-type lamins. Consistently, pRB Ser795 levels were increased in the *Lmna* ^+/+^-Gank cells and the *Lmna* ^−/−^ cells. Also p53 levels were decreased four-fold, consistent with previous reports showing that overexpression of gankyrin leads to p53 instability [41]. However, we did not observe a difference in p53 levels between *Lmna* ^−/−^ cells and *Lmna* ^+/+^-Cont cells (**Figure 3A**). To test if the *Lmna* ^+/+^-Gank cells have insufficient pRB function to maintain proper cell cycle regulation, we introduced GFP-p16^ink4a^ and examined the proliferative state of the cells by scoring BrdU incorporation into DNA over a 6 hr incubation period. p16^ink4a^-induced cell cycle arrest is known to be dependent on functional pRB [23]. We previously determined that the *Lmna* ^−/−^ cells were refractory to p16^ink4a^-mediated cell cycle arrest as a consequence of reduced pRB levels [22]. As opposed to *Lmna* ^+/+^-Cont cells, which have a 25% reduction of BrdU positive cells when transfected with GFP-p16^ink4a^ compared to cells transfected with GFP, the levels of BrdU are unchanged in *Lmna* ^+/+^-Gank cells, similar to *Lmna* ^−/−^ cells [22] (**Figure 3B**). These results suggest that gankyrin overexpression causes a physiologically relevant reduction in pRB levels in immortalized *Lmna^+/+^* fibroblasts.

Another CDK inhibitor, p21^cip1^, arrests cells in a *RB*-independent manner [42]. If *Lmna* ^+/+^-Gank cell cycle dysregulation is attributable to a loss of pRB function, then these cells should still undergo p21^cip1^-mediated cell cycle arrest. Consistently, we found that *Lmna* ^+/+^-Gank cells arrest normally in response to p21^cip1^, illustrating that gankyrin overexpressing cells behave similarly to *Lmna* ^−/−^ cells and are not resistant to all G1 arrest signals (**Figure 3B**).

To confirm that gankyrin overexpression impedes cell cycle progression, we conducted flow cytometric analysis on selected GFP positive cells from GFP-p16^ink4a^-transfected cells. As expected, p16^ink4a^ confers a 32% increase in the G1-phase cells and a 25% reduction in S-phase cells in *Lmna* ^+/+^-Cont cells, while the *Lmna* ^+/+^-Gank cells remain unresponsive to p16^ink4a^ overexpression (**Figure 3C**).

### Reduced expression of gankyrin in cells lacking A-type lamins does not restore pRB levels or function

**Figure 4:**
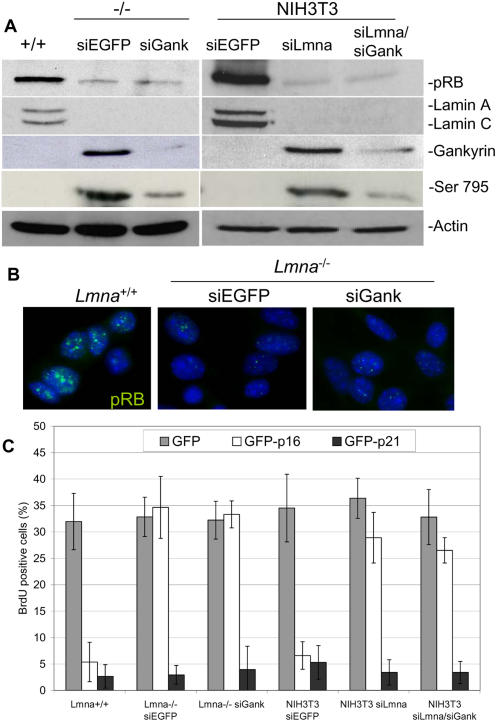
Reduction of gankyrin in cells lacking lamin A/C does not restore pRB or p16^ink4a^ sensitivity. Cells lacking lamin A/C (*Lmna* ^−/−^ and NIH3T3 siLmna cells) were infected with siRNAs targeted to gankyrin (siGank) or siRNAs targeted to a control gene, enhanced GFP (siEGFP). (A) Western blot analysis comparing cells lacking lamin A/C and gankyrin (*Lmna* ^−/−^-siGank and siLmna/siGank) to cells only lacking lamin A/C. (B) Indirect immunofluorescence of *Lmna* ^−/−^-siGank, *Lmna* ^−/−^-siEGFP cells, and litter-mate control *Lmna* ^+/+^ cells. Images comparing the cells were collected using equal exposure times and image processing. (C) Cells were transfected with GFP-p16^ink4a^ or GFP-p21^cip1^ and the S-phase cells were identified by BrdU incorporation. The percentage of cells positive for GFP and BrdU incorporation were quantified.

If increased levels of gankyrin in cells lacking lamin A/C are responsible for pRB instability, then pRB levels and function should be restored if gankyrin levels are decreased. To test this theory, we used siRNAs to reduce gankyrin expression in *Lmna* ^−/−^ cells (*Lmna* ^−/−^-siGank), achieving a ten-fold reduction compared to siRNAs targeted to a control gene, enhanced GFP (*Lmna* ^−/−^-siEGFP) (**Figure 4A**). The reduction of gankyrin reduced pRB phosphorylation at Ser 795 five-fold reduction indicating that gankyrin function was significantly impaired. Even though gankyrin expression was reduced and its activity significantly decreased, the total pRB levels remained unchanged in *Lmna* ^−/−^-siGank cells, similar to *Lmna* ^−/−^-siEGFP cells (**Figure 4A**). Consistently, by indirect immunofluorescence, the intensity of pRB nuclear foci was dramatically reduced in *Lmna* ^−/−^ siGank and *Lmna* ^−/−^-siEGFP cells compared to *Lmna* ^+/+^ cells (**Figure 4B**). To confirm that *Lmna* ^−/−^-siGank have insufficient pRB function to mediate cell cycle arrest, GFP-p16^ink4a^ was introduced and BrdU incorporation was monitored. Both *Lmna* ^−/−^-siGank and *Lmna* ^−/−^-siGFP cells remained refractory to p16^ink4a^-mediated cell cycle arrest (**Figure 4C**). One possible explanation for this finding is that the *Lmna^−/−^* cells used for these studies have been immortalized and may have mutations and/or altered expression in other genes that promote pRB destabilization (e.g. *MDM2*). To test this possibility we used siRNAs to reduce gankyrin expression in siLmna cells (siLmna/siGank cells). Although we were able to reduce both gankyrin and lamin A/C levels seven and ten-fold, respectively, and pRB phosphorylation at Ser795 was decreased five-fold, total pRB levels were still five-fold less than the siEGFP control cells (**Figure 4A**). We also verified that siLmna/siGank cells remained resistant to p16^ink4a^-induced arrest, and found no significant change compared to siLmna cells (**Figure 4C**). Overall, reduction of gankyrin in two cell lines lacking A-type lamins did not lead to enhanced pRB levels or restoration of p16^ink4a^ sensitivity suggesting that gankyrin is not solely involved in lamin A/C stability of pRB. We cannot completely rule out a role for gankyrin in these cells, since residual levels of the protein may be sufficient to mediate pRB degradation in siGank cells. A complete knockout of gankyrin would be necessary to examine this possibility. Another possibility is that loss of lamin A/C leads to degradation of pRB through multiple pathways one of which being gankyrin-dependent (see Discussion).

### MDM2 levels are increased in immortalized Lmna^−/−^ cells, but not siLmna cells

MDM2 was previously shown to stimulate pRB degradation in a proteasome-dependent manner [36], [37]. Increased MDM2 levels and/or activity in *Lmna^−/−^* cells may therefore be another avenue toward proteasome-dependent pRB degradation. To examine this possibility, we determined MDM2 levels in cells lacking lamin A/C. Again and surprisingly, immortalized *Lmna* ^−/−^ cells had six-fold higher MDM2 levels (**Figure 1A**). However, unlike gankyrin, MDM2 levels were not elevated in siLmna cells. One explanation for these disparate findings is that elevated MDM2 levels in *Lmna^−/−^* immortalized fibroblasts is an indirect consequence of the immortalization process and not related to loss of lamin A/C. It is possible that MDM2 may be required for pRB degradation even though its levels were not increased. Consequently, we used siLmna cells to test the importance of MDM2 for pRB destabilization in cells lacking lamin A/C.

### Disruption of the MDM2-pRB interaction does not restore pRB levels and function in siLmna cells

**Figure 5:**
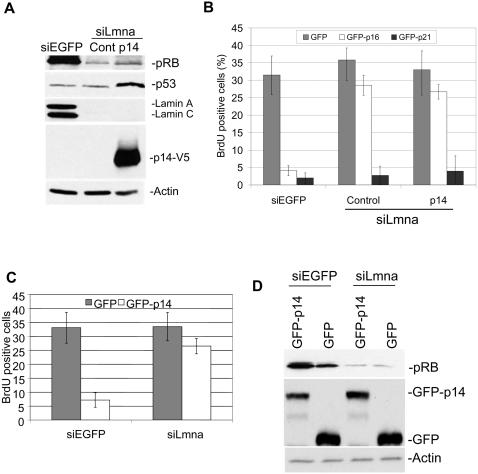
Expression of p14^arf^ in siLmna cells does not restore pRB levels or p16^ink4a^ sensitivity. NIH3T3 siLmna cells were stably transduced with a V5 tagged p14arf (siLmna-p14) or an empty vector (siLmna-Cont). (A) Western blot analysis comparing pRB levels in siEGFP cells, siLmna-Cont, and siLmna-p14. (B) Cells were transfected with GFP-p16ink4a or GFP-p21cip1 and the S-phase cells were identified by BrdU incorporation. The percentage of cells positive for GFP and BrdU incorporation were quantified. (C) siEGFP and siLmna cells were transfected with GFP-p14arf and the S-phase cells were identified by BrdU incorporation. (D) siEGFP and siLmna cells were transfected with GFP-p14arf and the GFP positive cells were sorted using FACS analysis.

To determine if MDM2 is involved in pRB stability in siLmna cells, we prevented pRB from interacting with MDM2 in siLmna cells. Previous studies have shown that disrupting the interaction between MDM2 and pRB results in loss of pRB ubiquitination and degradation, and subsequently increases pRB levels [43]. If MDM2 is responsible for pRB degradation in cells lacking lamin A/C, blocking the interaction between pRB and MDM2 should restore pRB function and levels. To test this theory we generated a siLmna cell line that stably expressed a V5 tagged p14^arf^ (siLmna-p14). p14^arf^ is a well characterized CDK inhibitor that inhibits MDM2 function and can prevent the interaction between MDM2 and pRB, thus causing accumulation of pRB [43]. Western analysis showed that pRB levels in siLmna- p14 cells were similar to the control infected cells (siLmna-Cont) and seven-fold reduced compared to the control siEGFP cells (Figure 5A). Consistently, siLmna-p14^arf^ cells remained refractory to p16^ink4a^ suggesting that the cells had insufficient pRB function to regulate the cell cycle (Figure 5B).

To verify that ectopic expression of p14^arf ^was functionally active, we analyzed p53 levels in the siLmna-p14 cells. According to previous reports, overexpression of p14^arf^ stabilizes p53 by preventing MDM2 from interacting with p53 [44]. Consistently, overexpression of p14^arf^ increased p53 levels four-fold in siLmna-p14 cells compared to siLmna-Cont cells indicating that p14^arf^ inhibited MDM2 activity (**Figure 5A**). In addition, we introduced GFP-p14^arf^ by transient transfection into siEGFP and siLmna cells and examined the proliferative state of the cells by scoring BrdU incorporation in GFP positive cells. Previous reports indicated that overexpression of p14^arf^ increased pRB levels and induced G1 arrest in a pRB dependent manner [43]. Consistently, GFP-p14^arf^ reduced the percentage of S-phase cells by 25% compared to the GFP transfected siEGFP cells, while BrdU incorporation in siLmna cells was unaffected (**Figure 5C**). Extracts of flow-sorted GFP positive cells were prepared and analyzed for pRB levels. Western analysis showed that GFP-p14^arf^ overexpression increased pRB levels two-fold in siEGFP cells, while siLmna cells were resistant to increased pRB levels by GFP-p14^arf^ overexpression (**Figure 5D**).

### Overexpression of p14^arf^ in NIH3T3 cells with reduced lamin A/C and gankyrin does not restore pRB levels or p16^ink4a^ sensitivity

**Figure 6:**
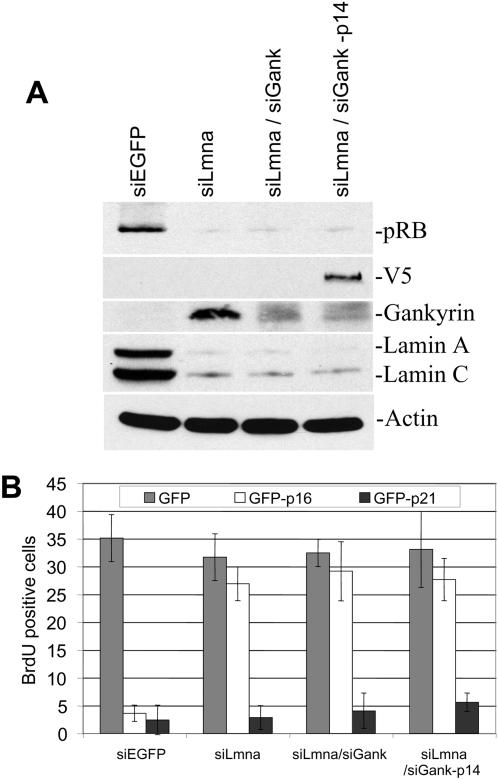
Expression of p14^arf^ in siLmna/siGank cells does not restore pRB levels or p16^ink4a^ sensitivity. NIH3T3 cells were infected with siRNAs targeted to *Lmna* and *Gankyrin* and then stably transduced with a V5 tagged p14^arf^ (siLmna/siGank-p14). (A) Western blot analysis comparing pRB levels in siEGFP cells, siLmna, siLmna/siGank, and siLmna/siGank-p14. (B) Cells were transfected with GFP-p16^ink4a^ or GFP-p21^cip1^ and the S-phase cells were identified by BrdU incorporation. The percentage of cells positive for GFP and BrdU incorporation were quantified.

Individually, we found no evidence indicating that gankyrin and MDM2 are responsible for pRB degradation in cells lacking lamin A/C. One possible explanation for these findings is that there is some functional redundancy between gankyrin- and MDM2-dependent pathways. Therefore, inhibiting the function of one protein might be insufficient to restore pRB levels and function. To determine if pRB levels could be restored when both gankyrin and MDM2 functions were deregulated, we stably transduced V5 tagged p14^arf^ in NIH3T3 cells with reduced lamin A/C and gankyrin expression (siLmna/siGank-p14). Western analysis demonstrated that interference of both MDM2 and gankyrin was insufficient to stabilize pRB (**Figure 6A**). Consistently, siLmna/siGank-p14 cells remained resistant to p16^ink4a^-induced arrest, indicating that reducing gankyrin levels and preventing the MDM2 and pRB association together were not sufficient to restore pRB levels in cells lacking lamin A/C (**Figure 6B**).

## Discussion

Gankyrin and MDM2 can both mediate degradation of the retinoblastoma protein [25], [Bibr pone.0000963-Maller1] [Bibr pone.0000963-Sdek1] [35]–[37]. Given that we have shown that pRB undergoes enhanced proteasome-dependent degradation in *Lmna^−/−^* cells, we determined whether gankyrin and MDM2 have any involvement in this process. We discovered that gankyrin levels are significantly elevated in *Lmna^−/−^* fibroblasts; however, RNAi-mediated reduction in gankyrin levels is insufficient to restore normal pRB stability to *Lmna^−/−^* cells, leading us to consider other pathways regulating pRB stability. Inhibition of MDM2 activity by overexpression of p14^arf^ also fails to restore pRB stability to *Lmna^−/−^* cells, and fails to confer cell cycle arrest. Finally, to test the hypothesis that MDM2 and gankyrin degrade pRB through redundant pathways in the absence of lamin A/C, p14^arf^ was expressed in *^-^*cells with reduced gankyrin and lamin A/C levels, and pRB levels were determined. We found that, again, pRB levels were not increased. While we cannot completely exclude a model whereby gankyrin and MDM2 have some involvement in pRB degradation in the absence of lamin A/C, these experiments lead us to the hypothesis that a third gankyrin-independent, MDM2-independent pathway to pRB degradation is invoked when A-type lamin function is ablated.

The mechanism(s) in which pRB becomes targeted for proteasome-dependent degradation remains to be well defined. However, a few other proteins, in addition to gankyrin and MDM2, have been linked to the pRB degradation [33]. For instance, viral oncoproteins such as human papillomavirus (HPV) E7, human cytomegalovirus (CMV) pp71, and Epstein-Barr virus nuclear antigen 3C (EBNA3C) all lead to pRB destabilization when expressed in mammalian cells [45]–[50]. The pathway(s) by which pRB becomes degraded in the presence of viral proteins remains to be fully elaborated. For instance, viral proteins have been reported to target pRB for degradation through both proteasome-dependent and independent mechanisms [46], [47], [51]. In addition to pRB, Knight et al. EBNA3C interacts with the SCF ubiquitin ligase, suggesting a role for this complex in pRB degradation [49]. Consistently, the expression of a dominant negative Skp2 restored pRB stability in EBNA3C-expressing cells. These findings raise the possibility that pRB undergoes increased exposure to SCF in the absence of lamin A/C leading to its degradation through this ligase system.

Overexpression of the acetyltransferase Tip60 increases pRB acetylation and targets the tumor suppressor for proteasome-dependent degradation [52]. Tip60 is a component of the NuA4 complex that participates in transcriptional activation and the DNA damage response [53]. We considered the possibility that increased Tip60-dependent acetylation of pRB might be a contributing factor toward pRB degradation in *Lmna^−/−^* cells, but believe this unlikely since increased p14^arf^ expression is known to counteract this effect [52], and yet p14^arf^ was unable to restore pRB levels in cells lacking lamin A/C. Nevertheless, potential changes in pRB acetylation associated with loss of A-type lamins should be determined.

### Lamin A/C is important in regulating gankyrin transcription

Gankyrin is a relatively unknown protein that was discovered within the last ten years. The majority of the gankyrin research has involved its ability to stimulate proteasome-dependent degradation of pRB and p53, and its role in pRB phosphorylation [27], [32]. Very little information is known about the mechanism of gankyrin regulation. Gankyrin was identified by increased transcript levels in hepatocellular carcinomas (HCCs) indicating that *gankyrin* might be regulated at the transcriptional level [25]. However, no specific studies have analyzed gankyrin expression. By analyzing cell lines that are deficient in lamin A/C, we discovered that in fibroblasts *gankyrin* transcript levels are increased four to five-fold and gankyrin protein levels are increased seven-fold in the absence of A-type lamins (**Figure 1B**). While we cannot rule out a model whereby *Lmna^−/−^* cells have increased *gankyrin* mRNA stability and not transcription, our findings add to the growing evidence that lamin A/C, either directly or indirectly, is important for regulating a subset of genes at the transcriptional level [14], [20], [54]–[56].

Interestingly, altered transcription of gankyrin in the absence of lamin A/C, appears to be tissue-specific. QPCR analysis of myoblasts lacking lamin A/C indicated that, unlike fibroblasts gankyrin transcript levels are not increased (**Figure S2**). The reasons for this tissue-specificity are not known and it would be interesting to examine the expression of gankyrin in a variety of tissues from *Lmna^−/−^* mice, particularly since increased gankyrin expression is associated with hepatocarcinogenesis [25], [26].

### Lamin A/C and tumorigenesis

Reduced lamin A/C expression is a common feature of a variety of different cancers [39], however there is little direct evidence connecting tumorigenesis to A-type lamins. Through our previous work we showed that decreased expression of lamin A/C results in reduced activity of a known tumor suppressor, pRB [21], [22]. This reduction was functionally significant since *Lmna^−/−^* cells, like *Rb^−/−^* cells [23], are refractory to p16^ink4a^-mediated cell cycle arrest [38]

Findings in this report further support a role for A-type lamins as potential tumor suppressors. In addition to having increased gankyrin protein levels, we find that *Lmna^−/−^* cells are refractory to p14^arf^-mediated cell cycle arrest. In addition to *RB*, *p16^ink4a^* and *p14^arf^* are linked to cellular senescence and are common targets for inactivation mutation during carcinogenesis [26]. Since neither of these key tumor suppressors impede cell cycle progression in *Lmna^−/−^* cells, it is more likely that mutations inactivating A-type lamin function also contribute to tumorigenesis.

## Materials and Methods

### Cell Culture and Plasmid transfection

Immortalized knockout fibroblast cell lines, as well as litter-matched controls, have been described [5]. All cell lines were cultured in Dulbecco's Modified Eagle Media (DMEM) supplemented with 10% fetal bovine serum (FBS), 2mM L-glutamine, 10 U/mL penicillin, 10 µg/ml streptomycin. Cells grown to 30% confluency were transfected using Lipofectamine PLUS (Invitrogen). The following constructs were used: pcDNA3.1 GFP, pcDNA3.1-GFP- p16^ink4a^, and pcDNA3.1-GFP-p21^cip1^. To determine the percentage of S-phase cells, cells were grown on coverslips for 36 hours, pulsed with BrdU for 6 hours, then fixed with 4% paraformaldehyde and detected by immunofluorescence as previously described [23]. Results were recorded from triplicate samples, and 150 cells were scored per sample. Each experiment was repeated three times and the data presented is a representative sample.

### Retroviral infections

The retroviruses pMXIH-V5, pMXIH-gankyrin-V5, pMXIH-p14^arf^-V5, and pMXIH-human lamin A-res were used for infections [57]. The human lamin A-res plasmid was generated as described in Frock et al. 2006 [20]. 36 hours after infection, infected cells were selected by culturing in selective media containing 500 µg/mL of hygromycin-B for 2 days. The retroviruses pLXSN and pLXSN- p14^arf^ (kind gifts from Marie Classon Cancer Center, Massachusetts General Hospital) were infected into cells and were selected for ten days using 600 µg/ml G418. 24 hours before additional transfection, the selective media was removed from the infected cells and supplemented DMEM (described above) was added. Infection of retroviral small inhibitory RNA (siRNA) constructs to mouse gankyrin was conducted as previously described [57]. The siRNA sequence for mouse gankyrin is 5′-GCCTGGGTTTAATACTCAA -3′. Ecotropic retroviruses of pSuper.retro-Puro (Oligoengine) and pSuper.retro-Puro siLamin A plasmids was generated as described above. As a control siRNAs targeted to enhanced GFP were used [57]. 24 hours after NIH3T3 cells were infected, cells were selected using 5 µg/mL of puromyocin for 3 days. These cells were not used for experiments more than 6 passages after selection.

### Indirect Immunofluorescence

Immunofluorescence was performed on formaldehyde-fixed cells except for those stained for lamin A/C (SC-20681), which were methanol-fixed as previously described [5]. The following antibodies were used: mouse anti-pRB (Pharmingen, G3-245) and mouse anti-BrdU (Becton Dickinson, 347580). Images were taken using Zeiss Axiovert 200 (Obserkochen, Germany). Images that compared protein levels were collected using equal exposure times and processed similarly.

### Protein Analysis

Protein extracts from total cells were harvested in RIPA buffer (0.15 M NaCl/0.05mM Tris-HCl, pH 7.2/1% NP-40/1% sodium deoxycholate/0.1% SDS). A total of 40 to 50 µg of total protein was separated by denaturing electrophoresis. The following antibodies were used for immunoblotting: mouse anti-pRB (Pharmingen, G3-245), rabbit anti-pRB Ser795 (Cell Signaling, 9301), rabbit anti-pRB Ser807/811 (Cell Signaling, 9308), mouse anti-actin (Chemicon, MAB1501R), rabbit anti-MDM2 (Santa Cruz, H221), anti-mouse V5 (Invitrogen, R960-25), mouse anti-p53 (Cell Signaling, 1C12), rabbit anti-gankyrin (Santa Cruz, H-231), rabbit anti-pan lamin A/C (Cell Signaling, 2032), and mouse anti-GFP (Roche, 11814460001). Protein levels were determined by counting pixels using NIH Image (National Institute of Health, USA).

### Fluorescent Activated Cells Sorting (FACS) analysis

36 hours after transfection, flow cytometry analysis was conducted on the GFP transfected cells in the Rabinovitch Laboratory (Dept Pathology, University of Washington). Cells were washed in a solution of 10 µg/ml 4,6-diamidino-2-phenylindole (DAPI) and 0.1% nonidet P-40 detergent in a Tris buffered saline and then triturated with a 26 gauge needle and analyzed using a Coulter ELITE cytometer (Coulter Corp., Miami FL), with ultraviolet excitation and DAPI emission collected at >450nm [58]. DNA content and cell cycle were analyzed using the software program WinCycle software (Phoenix Flow Systems, San Diego, CA) as previously described [59]. At least 25,000 cells were counted per experiment. The data are presented as histograms in which cell number is plotted against DNA content. Cells were sorted by GFP expression using a similar manner. Approximately 20,000 cells were collected per sample and analyzed using western analysis.

### Quantitative PCR (QPCR) analysis

mRNA's were purified using the RNeasy kit (Qiagen). For QPCR, TaqMan One-Step RT-PCR was mixed with 50 ng of RNA and to generate cDNA (Applied Biosystems) and 300 µM forward and reverse primers. Triplicates of the cDNA's were amplified on the Opticon I real-time thermal cycler (MJ Research). The experiments were performed three times. PCR products were normalized against the housekeeping gene *Gapdh*, and measurements between samples were compared by cycle threshold (Ct). Primer sequences used for QPCR for the mouse gankyrin (Mm00450376_m1)) and mouse *Gapdh* (FAM-MGB) were obtained from Applied Biosystems.

## Supporting Information

Figure S1 Overexpression of gankyrin leads to pRB degradation by the proteasome in U2OS cells. U2OS cells were stably transduced with gankyrin-V5 or a vector control and were treated with a proteasome inhibitor (MG132) and/or cycloheximide (CHX) for the specified times. Protein samples were separated by a 8% SDS PAGE and detected by immunoblot with mouse anti-pRB. The percent of pRB was determined by counting pixel levels using NIH Image and each time point was normalized to time 0 hr. Actin was used as a loading control.
(6.38 MB TIF)

Figure S2 QPCR analysis comparing the mRNA's of gankyrin in Lmna-/- myoblasts. -/- cells are derived from the Lmna-/- mouse. +/+ cells are the litter-mate control cells, which possess endogenous lamin A/C. siLmna cells are NIH3T3 cells with siRNAs targeted to Lmna. siGFP cells are NIH3T3 cells with siRNAs targeted to a control gene, enhanced GFP. Gankyrin mRNA's are increased four-fold in Lmna-/- fibroblastss and siLmna cells, compared to the control cells. Lmna-/- myoblasts possess similar gankyrin mRNA levels as Lmna+/+ myoblasts. Data represent averages of triplicate experiments performed and mRNAs were normalized against Gapdh.
(6.38 MB TIF)
