# Supplementary figures and images for: Evidence that Proteasome-Dependent Degradation of the Retinoblastoma Protein in Cells Lacking A-Type Lamins Occurs Independently of Gankyrin and MDM2
Source: PLoS One. 2007 Sep 26;2(9):e963. doi: 10.1371/journal.pone.0000963 (PMC1978514; doi:10.1371/journal.pone.0000963)

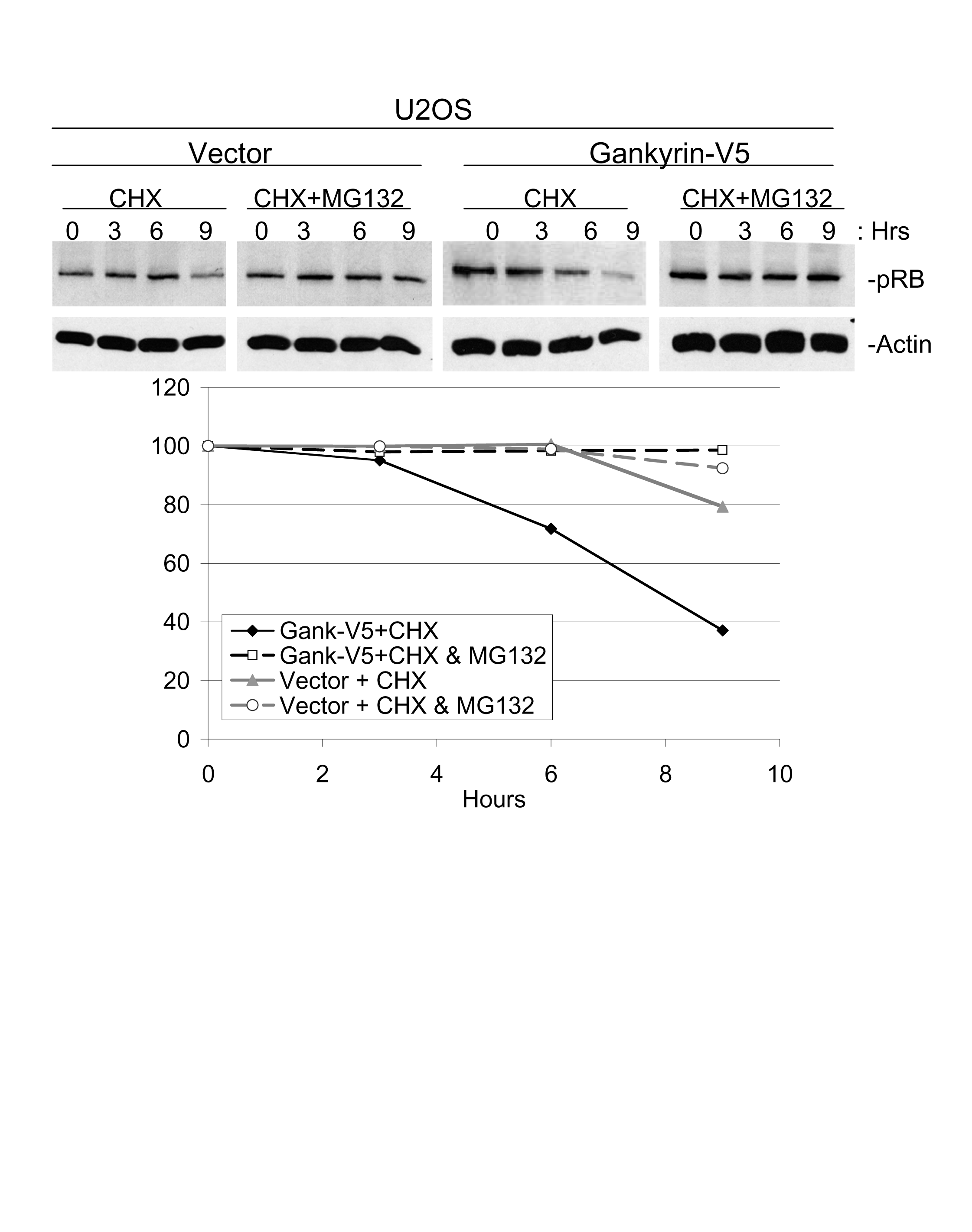

Supplement: Figure S1 — Overexpression of gankyrin leads to pRB degradation by the proteasome in U2OS cells. U2OS cells were stably transduced with gankyrin-V5 or a vector control and were treated with a proteasome inhibitor (MG132) and/or cycloheximide (CHX) for the specified times. Protein samples were separated by a 8% SDS PAGE and detected by immunoblot with mouse anti-pRB. The percent of pRB was determined by counting pixel levels using NIH Image and each time point was normalized to time 0 hr. Actin was used as a loading control. (6.38 MB TIF) [file pone.0000963.s001.tif]

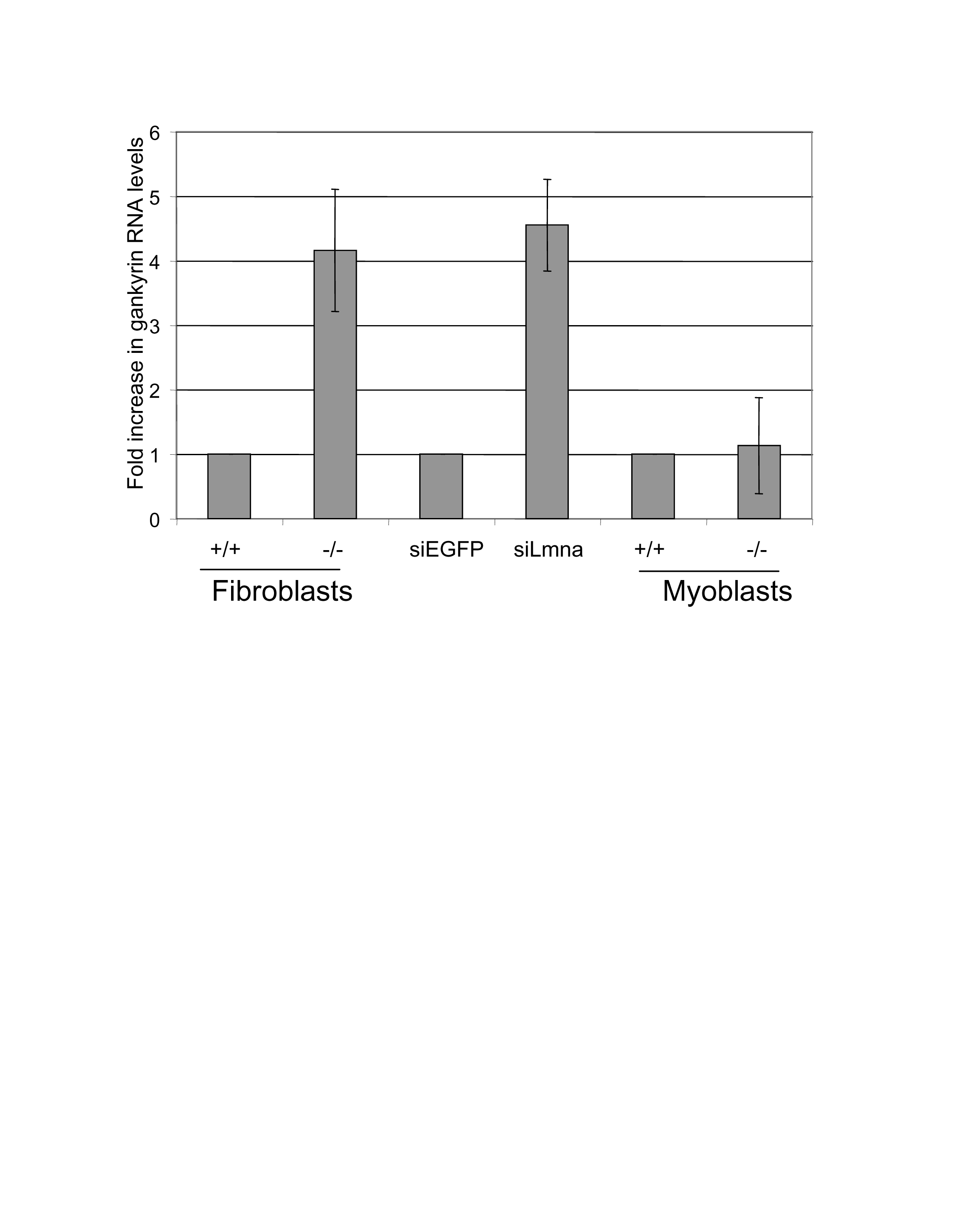

Supplement: Figure S2 — QPCR analysis comparing the mRNA's of gankyrin in Lmna-/- myoblasts. -/- cells are derived from the Lmna-/- mouse. +/+ cells are the litter-mate control cells, which possess endogenous lamin A/C. siLmna cells are NIH3T3 cells with siRNAs targeted to Lmna. siGFP cells are NIH3T3 cells with siRNAs targeted to a control gene, enhanced GFP. Gankyrin mRNA's are increased four-fold in Lmna-/- fibroblastss and siLmna cells, compared to the control cells. Lmna-/- myoblasts possess similar gankyrin mRNA levels as Lmna+/+ myoblasts. Data represent averages of triplicate experiments performed and mRNAs were normalized against Gapdh. (6.38 MB TIF) [file pone.0000963.s002.tif]
